# Supplementary material for: Reduction of Blood Oxidative Stress Following Colorectal Cancer Resection
Source: Cancers (Basel). 2024 Oct 21;16(20):3550. doi: 10.3390/cancers16203550 (PMC11505646; doi:10.3390/cancers16203550)
Supplement: Supplementary file 1 [file cancers-16-03550-s001.zip › cancers-3239520-supplementary.pdf]

Figure S1 Scatter plot of d-ROMs and BAP values after surgery

d-ROM

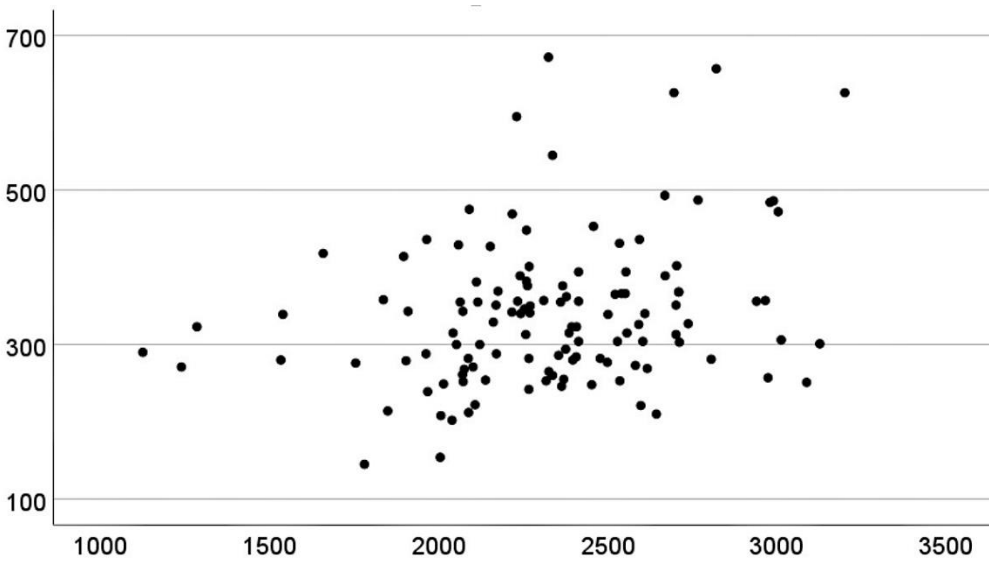

BAP

Spearman's correlation coefficient: 0.187

FigureS2 Changes in d-ROM Before and After Surgery by Stage

FigureS2a Changes in postoperative BAP(123cases)

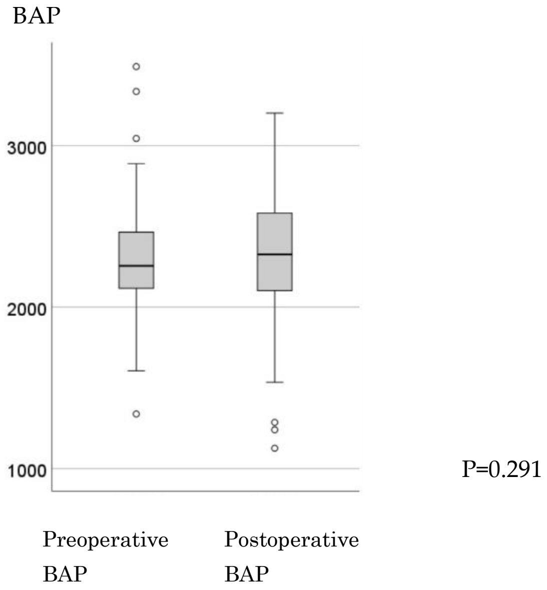

Figure S2b Changes in postoperative BAP(stage0,1)

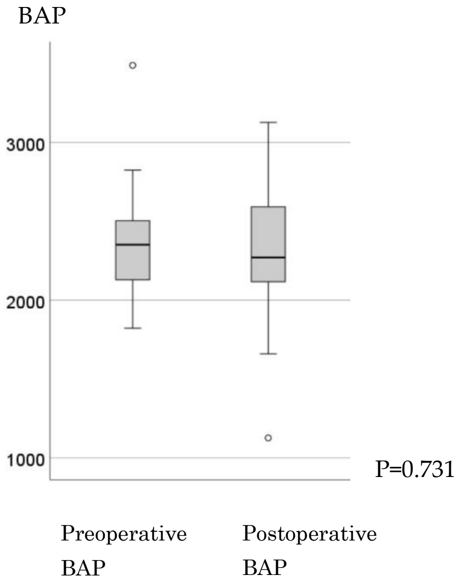

Figure S2c Changes in postoperative BAP(stage2)

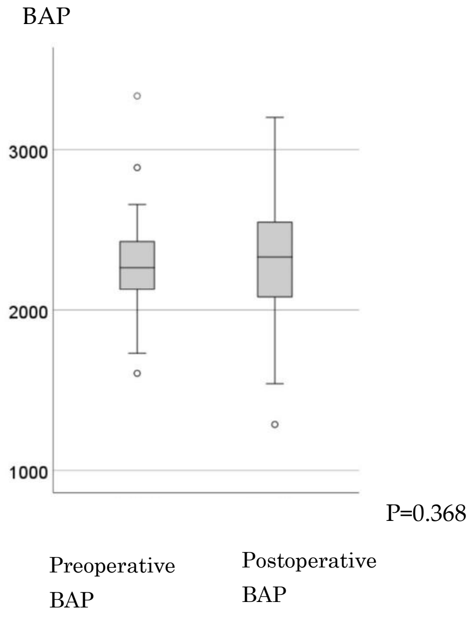

Figure S2d Changes in postoperative BAP(stage3)

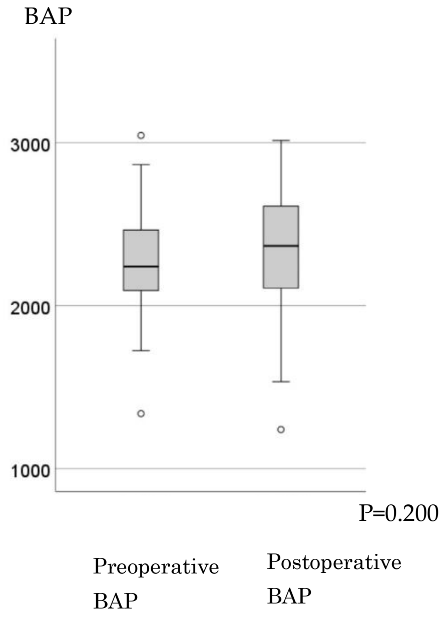

Table S1

Comparison of postoperative d-ROM and BAP values with clinicopathological factors in colorectal cancer patients using the Mann-Whitney U test.

Independent variables	case	d-ROMs Median (IQR)	d-ROMs p-value	BAP Median (IQR)	BAP p-value
Age (years)			0.972		0.972
<70	54	331.0 (271.5, 392.8)		2327.5 (2079.3, 2615.3)	
≥70	69	327.0 (282.0, 369.0)		2326.0 (2116.0, 2535.0)	
Sex			<0.001		0.696
Male	66	303.5 (257.8, 54.0)		2331.0 (2079.3, 2577.8)	
Female	57	356.0 (300.0, 402.0)		2326.0 (2108.0, 2557.0)	
Location			0.992		0.554
Right	19	319.0 (280.0, 370.8)		2373.0 (2115.0, 2545.0)	
Left	104	329.0 (272.0, 379.0)		2271.0 (2074.0, 2587.0)	
Tumor size (mm)			0.726		0.961
<45	75	339.0 (278.0, 385.5)		2325.0 (2090.0, 2544.5)	
≥45	48	323.0 (279.0, 360.5)		2346.0 (2119.5, 2602.8)	
Tumor invasion depth			0.110		0.450
T1, T2	42	349.0 (285.5, 424.8)		2343.5 (2155.3, 2593.5)	
T3, T4	81	315.0 (273.0, 365.0)		2325.0 (2076.0, 2552.0)	
Lymph node metastasis			0.161		0.814
No	81	341.0 (281.5, 391.5)		2318.0 (290.0, 2569.5)	
Yes	42	314.0 (275.5, 356.3)		2367.0 (2111.0, 2566.5)	

Stage			0.084		0.904
0-I	41	355.0 (284.0, 418.0)		2270.0 (2116.0, 2592.0)	
II-III	82	315.0 (273.8, 357.8)		2346.0 (2088.3, 2553.5)	

Table S2 Multivariate linear regression analysis results for postoperative d-ROM levels in patients with colorectal cancer

Independent variables	Multivariate linear regression analysis		
	β	e^{β} (95%CI)	p-value
Age	1.11 e^{-3}	1.001 (5.34e^{-3}, 2.14e^{-3})	0.606
Sex (men vs. female)	1.58 e^{-1}	1.171 (6.00e^{-2}, 2.57e^{-1})	0.182
Location (left vs. right)	3.24 e^{-2}	1.033 (-7.20e^{-2}, 1.37e^{-1})	0.540
Tumor size	1.20 e^{-3}	1.001 (-1.88e^{-3}, 4.27e^{-3})	0.442
Serosa invasion (no vs. yes)	-4.54 e^{-2}	0.956 (-1.70e^{-1}, 7.93e^{-2})	0.472
Lymph node metastasis (no vs. yes)	-4.76 e^{-2}	0.954 (-1.60e^{-2} ~6.56e^{-2})	0.407

β: regression coefficient CI: confidence interval

Table S3 Multivariate linear regression analysis results for postoperative d-ROM levels in patients with colorectal cancer

Independent variables	Multivariate linear regression analysis		
	β	e^{β} (95%CI)	p-value
Age	8.53 e^{-4}	1.001 (-2.03e^{-3}, 3.74e^{-3})	0.559
Sex (men vs. female)	2.30 e^{-2}	1.023 (-4.38e^{-2}, 8.99e^{-2})	0.496
Location (left vs. right)	-3.61 e^{-3}	0.996 (-7.47e^{-2}, 6.75e^{-2})	0.920
Tumor size	-1.97 e^{-4}	1.000 (-2.29e^{-3}, 1.89e^{-3})	0.852
Serosa invasion (no vs. yes)	-2.92 e^{-2}	0.971 (-1.14e^{-1}, 5.57e^{-2})	0.497
Lymph node metastasis (no vs. yes)	2.76 e^{-2}	1.028 (-4.93e^{-2} ~1.04e^{-1})	0.400

β: regression coefficient CI: confidence interval

Table S4 Multivariate linear regression analysis results for BAP ratio in patients with colorectal cancer

Independent variables	Multivariate linear regression analysis		
	β	95%CI	p-value
Age	1.99 e^{-3}	-1.41e^{-3}, 5.40e^{-3}	0.248
Sex (men vs. female)	3.42e^{-2}	-4.47e^{-2}~ 1.13e^{-1}	0.392
Location (left vs. right)	2.17	-6.23e^{-2}, 1.06e^{-1}	0.610
Tumor size	-7.90e^{-4}	-3.26e^{-3}, 1.68e^{-3}	0.527
Serosa invasion (no vs. yes)	7.17e^{-3}	-9.30e^{-2}, 1.07e^{-1}	0.888
Lymph node metastasis (no vs. yes)	4.08e^{-2}	-1.41e^{-3} ~5.40e^{-3}	0.376

β: regression coefficient   CI: confidence interval
